# Functions of *S*-nitrosylation in plant hormone networks

**DOI:** 10.3389/fpls.2013.00294

**Published:** 2013-08-01

**Authors:** Ramiro Parí, Marí J. Iglesias, Marí C. Terrile, Claudia A. Casalongué

**Affiliations:** Instituto de Investigaciones Biológicas, Facultad de Ciencias Exactas y Naturales, Unidade Ejecutora-Consejo Nacional de Investigaciones Cientïficas y Técnicas - Universidad Nacional de Mar del PlataMar del Plata, Argentina

**Keywords:** nitric oxide, phytohormones, redox mechanism, signaling, *S*-nitrosylation

## Abstract

In plants, a wide frame of physiological processes are regulated in liaison by both, nitric oxide (NO) and hormones. Such overlapping roles raise the question of how the cross-talk between NO and hormones trigger common physiological responses. In general, NO has been largely accepted as a signaling molecule that works in different processes. Among the most relevant ways NO and the NO-derived reactive species can accomplish their biological functions it is worthy to mention post-translational protein modifications. In the last years, *S*-nitrosylation has been the most studied NO-dependent regulatory mechanism. Briefly, *S*-nitrosylation is a redox-based mechanism for cysteine residue modification and is being recognized as a ubiquitous regulatory reaction comparable to phosphorylation. Therefore, it is emerging as a crucial mechanism for the transduction of NO bioactivity in plants and animals. In this mini-review, we provide an overview on *S*-nitrosylation of target proteins related to hormone networks in plants.

## INTRODUCTION

Nitric oxide (NO) is a free-radical product of cell metabolism, being nitrate reductase the best characterized enzymatic pathway for NO production in plants. However, other reductive and oxidative routes have been also described (Lamattina and Polacco, 2007). It functions as a ubiquitous signal involved in diverse physiological processes and it is frequently implicated in multiple cell signaling events under the control of phytohormones including growth, development, and stress responses. Nevertheless, in most cases the molecular mechanisms underlying NO action in the plant cell are still undeciphered. The overlapping roles between plant hormones and NO raise the question of how both molecules may act in coordination. In general, regulatory effects of NO are mediated through protein modifications, including tyrosine nitration, metal nitrosylation, and *S*-nitrosylation of cysteines. Thus, the identification of NO primary targets has provided new opportunities to link NO reactivity and biological processes. In this review, we highlight the progress brought by the identification of *S*-nitrosylated target proteins related to stress and growth-promoting plant hormones. Our focus is the broad role of this post-translational modification that allows NO to modulate plant hormone homeostasis as well as signaling pathways. However, the participation of NO beyond its action through *S*-nitrosylation in hormone-regulated processes is out of the scope of this work and it is widespreadly covered in recent reviews by Simontacchi et al. (2013) and Astier and Lindermayr (2012).

## *S*-NITROSYLATION AS AN EMERGING POST-TRANSLATIONAL MODIFICATION OF PLANT PROTEINS

*S*-nitrosylation is the reversible binding of a NO moiety to a reactive cysteine residue of a target protein to form an *S*-nitrosothiol (SNO; Stamler et al., 2001). It is recognized as a reversible and ubiquitous regulatory reaction. Thus, like in animals, this redox-based post-translational mechanism is also crucial for the transduction of NO bioactivity in many plant cellular responses (Hess et al., 2005). At first, protein *S*-nitrosylation was thought to be controlled mainly through the regulation of NO biosynthesis. However, in mammals it has been postulated as a short-range NO post-translational mechanism limited to proximity of NO sources (Martinez-Ruiz et al., 2013). In addition to the enzymatic NO-producing enzymes, it is important to consider that both, favorable environment to *S*-nitrosylating agent formation as well as transnitrosylating reactions could promote the expansion of the *S*-nitrosylation range of action (Martinez-Ruiz et al., 2013). The SNO turnover could also provide an alternative mechanism to control protein *S*-nitrosylation in the cell. Given the labile nature of this post-translational modification, it was conceived initially as a spontaneous and non-regulated process. However, different denitrosylase enzymes have been described, which directly mediate denitrosylation or govern the cellular equilibrium between protein and low-molecular weight SNOs. Two main enzymatic systems have emerged as physiologically relevant denitrosylases: the glutathione/*S*-nitrosoglutathione reductase (GSH/GSNOR) and the thioredoxin/thioredoxin reductase (Trx/TrxR; Benhar et al., 2009). *S*-nitrosylation of the major intracellular antioxidant tripeptide GSH forms *S*-nitrosoglutathione (GSNO) that functions as a mobile reservoir of NO. Consequently, the enzyme GSNOR or GSNOR1 in *Arabidopsis* does not display a direct denitrosylase activity but controls intracellular levels of both, GSNO and SNO affecting the global level of *S*-nitrosylation (Feechan et al., 2005; Malik et al., 2011). On the other side, the mechanism described in animals for Trx denitrosylation involves direct interaction with SNO-proteins by formation of an intermolecular disulphide intermediate in which Trx is covalently linked to the substrate protein through a disulphide bridge, or transnitrosylation in which Trx is transiently *S*-nitrosylated (Benhar et al., 2009). Trx have been also described in the denitrosylation process taking part in hormonal signaling in plants (Tada et al., 2008). Therefore, it appears that the balance between *S*-nitrosylation/denitrosylation is critical for the precise transduction of NO signal.

*S*-glutathionylation is the post-translational modification of protein cysteine residues by the addition of GSH (Martinez-Ruiz and Lamas, 2007). The integrative interplay between protein *S*-glutathionylation and *S*-nitrosylation could be recognized as another crucial network for post-translational modification of certain proteins. Although the *S*-glutathionylation of proteins has been generally described more than 20 years ago, the identification of protein targets for this modification remains rather unexplored. Interestingly, for some mammal proteins involved in clinical disorders such as cardiovascular disease and diabetes among others, *S*-nitrosylation has been described as an intermediate for more stable modifications like *S*-glutathionylation (Martinez-Ruiz and Lamas, 2007). In summary, *S*-nitrosylation is crucial for NO signal transduction pathway but it should also be noted that other related-*S*-nitrosylation regulators can converge in NO-mediated protein functionality in plants.

## *S*-NITROSYLATION OF TARGET PROTEINS LINKED TO STRESS PHYTOHORMONES

Salicylic acid (SA) and ethylene (ET) are key signaling molecules for plants in the resistance to biotic stress (Fujita et al., 2006; Loake and Grant, 2007). NO has an essential role in restriction of pathogen attack by induction of the defense response and programed host cell death (reviewed by Mur et al., 2013). Thus, NO bioactivity may exert a role on SA and ET hormone signaling pathways.

**FIGURE 1:**
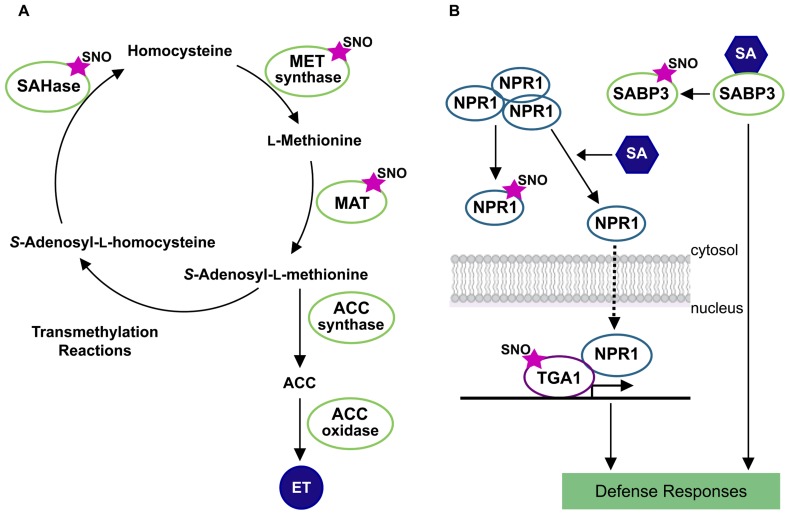
***S*-nitrosylation of target proteins in ethylene biosynthesis and salicylic acid network.** The figure shows a schematic representation of methylmethionine cycle in the ethylene (ET) synthesis **(A)** and salicylic acid (SA) signaling networks **(B)**. Protein *S*-nitrosothiols are represented by an SNO mark. References to physiological processes regulated by hormones, and subcellular localizations in the cell are also indicated. SAHase, adenosylhomocysteinase; MET synthase, cobalamin-independent methionine synthase; MAT, methionine adenosyltransferase; ACC, aminocyclopropane-1-carboxylic acid; SABP3, salicylic acid binding protein 3; NPR1, non-expresser of pathogenesis-related gene1 protein; TGA1, transcription factor TGA1.

In *Arabidopsis*, one of the first comprehensive proteomic studies allowed the identification of more than 100 *S*-nitrosylated proteins (Lindermayr et al., 2005). Interestingly, one of the identified *S*-nitrosylated proteins corresponded to a methionine adenosyltransferase (MAT) which catalyzes the synthesis of *S*-adenosylmethionine (SAM), a substrate for ET biosynthesis. Later on, Lindermayr et al. (2006) provided the first detailed molecular characterization of an *S*-nitrosylated target protein in plants. This study describes the *S*-nitrosylation of Cys-114 residue of the MAT1 isoform and the consequently inhibition of its activity. The enzymes *S*-adenosylhomocysteinase and cobalamin-independent methionine synthase are also part of the methylmethionine cycle and both enzymes have been found to be *S*-nitrosylated in proteomic analysis in *Arabidopsis* and *Kalanchoe pinnata* plants (Lindermayr et al., 2005; Abat et al., 2008). Activation/inactivation of these enzymes controls the SAM pool impacting in ET biosynthesis. All these evidences point out a multi-step control of ET biosynthesis by *S*-nitrosylation and opened the possibility to elucidate new mechanisms of NO and ET cross-talk (**Figure 1A**).

Salicylic acid is synthesized by plants in response to pathogen infection and is essential to the establishment of resistance mechanisms, including host cell death and systemic acquired resistance. Mutations in *AtGSNOR1* showed a pivotal role in the GSNO turnover, influencing cellular SNO levels under both, basal conditions and attempted microbial attack (Feechan et al., 2005). Interestingly, in the absence of AtGSNOR1 both SA biosynthesis and signaling are affected, suggesting that *S*-nitrosylation may control at least, two nodes of the SA-signaling network. GSNOR1 regulates the *S*-nitrosylation extent of non-expresser of pathogenesis-related gene1 (NPR1) and SA binding protein 3 (SABP3; Tada et al., 2008; Wang et al., 2009). *S*-nitrosylation of SABP3 is triggered during bacterial infection and suppresses SA binding capacity and carbonic anhydrase (CA) activity (Wang et al., 2009). Since, CA activity is required for the establishment of plant disease resistance, its inhibition by *S*-nitrosylation during late infection stages could contribute to a negative feedback loop which could be crucial for the proper modulation of SA-dependent plant defense mechanism (**Figure 1B**).

*S*-nitrosylation also exerts a key redox control of systemic acquired resistance in plants through targeting NPR1/TGA1 system. The SA NPR1-dependent signaling mechanism is mediated by redox changes that lead to reduction of NPR1 cysteines. This event switches NPR1 from cytosolic, disulfide-bound oligomers, to active monomers that are subsequently translocated into the nucleus and interacts with the TGA class of basic leucine zipper transcription factors. The result is an enhanced binding activity of TGA1 to the promoter region of pathogenesis-related (PR) genes, stimulating SA-dependent immune defense (Vlot et al., 2009). Upon pathogen attack, SA induces Trx which facilitates NPR1 monomerization, nuclear translocation, and activation of PR genes (Tada et al., 2008). Additionally, Tada et al. (2008) demonstrated that NPR1 is an *S*-nitrosylated protein. Notably, TGA1 is regulated by *S*-nitrosylation and *S*-glutathionylation improving TGA1 binding activity to PR1 promoter region (Lindermayr et al., 2010). However, it has not been demonstrated which type of modification, *S*-nitrosylation, *S*-glutathionylation, and/or both, is responsible for such protein–DNA binding activity (**Figure 1B**).

Concluding, plant immunity is regulated by a precise redox balance between the opposing actions of distinct redox-signals that catalyze NPR1 oligomer–monomer switch and NPR1/TGA1 interaction through transient redox fluctuations that includes *S*-nitrosylation and *S*-glutathionylation. Moreover, in the cytosol NPR1 also contributes to the suppression of jasmonic acid (JA)-dependent responses (Spoel et al., 2003), evidencing *S*-nitrosylation as a mediator of the integrative hormonal regulation network for guarantee immunity in plants.

Meanwhile, abscisic acid (ABA) is the major player mediating adaptation of plants to drought stress. ABA induces stomatal closure and inhibits stomatal opening by facilitating osmotic solute loss to reduce guard cell turgor. These events take place through a complex signaling network that involves multiple components including Ca^2^^+^, K^+^, IP_3_, MAPK, and H_2_O_2_ (Fan et al., 2004). NO enhances plant tolerance to drought and it contributes to stomatal closure evoked by ABA. Mechanistically, NO regulates inward-rectifying K^+^ channels through its action on Ca^2^^+^ release from intercellular stores. Alternative pathways have been also indicated for NO action on the outward-rectifying K^+^ channels, which are Ca^2^^+^ insensitive. It is probable that NO directly modifies the K^+^ channel at the guard cell plasma membrane or a closely associated regulatory protein through *S*-nitrosylation (Sokolovski and Blatt, 2004). However, the physiological significance of this regulation remains unexplored.

## TARGETS FOR PROTEIN *S*-NITROSYLATION IN SIGNALING PATHWAYS OF GROWTH-PROMOTING PHYTOHORMONES AUXINS AND CYTOKININS

**FIGURE 2:**
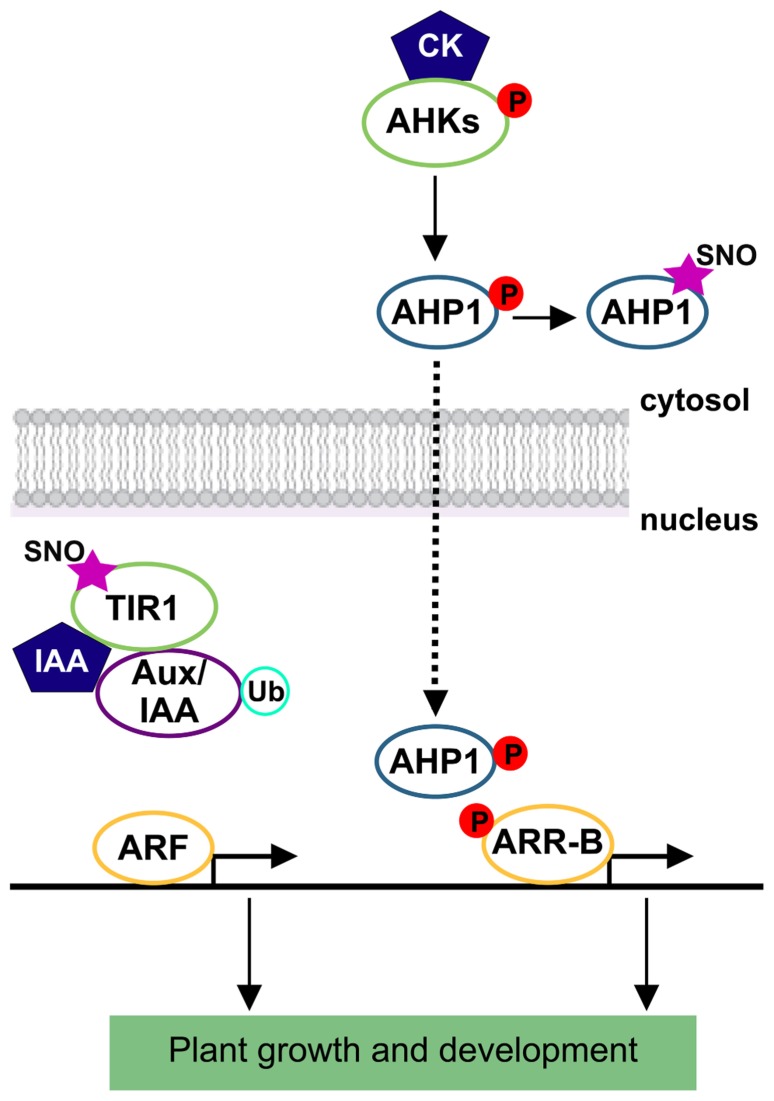
**Auxin and cytokinin signaling pathways are under protein *S*-nitrosylation influence.** Overview of indole-acetic acid (IAA) and cytokinin (CK) signaling pathways. *S*-nitrosothiols are represented by an SNO mark. Protein phosphorylation is represented by a P letter. References to physiological processes regulated by hormones, and subcellular localizations are also indicated. TIR1, transport inhibitor response 1 protein; Aux/IAA, auxin/indole-3-acetic acid protein; ARF, auxin response factor; Ub, ubiquitin; AHKs, hybrid histidine protein kinases; AHP1, histidine phosphotransfer protein 1; ARR-B, primary response regulator type B.

Auxins and cytokinins (CKs) are critical regulators of cell division, expansion, and differentiation.****Relatively recent breakthroughs were found by comparing functions of NO and the well-known growth-promoting hormones (reviewed by Mur et al., 2013). There are several examples of NO and auxin overlapping effects during shoot and root organogenesis such as, NO mediation of auxin-induced adventitious and lateral roots (Pagnussat et al., 2002; Correa-Aragunde et al., 2004), root hair formation (Lombardo et al., 2006), and adventitious root formation (Pagnussat et al., 2003). NO stimulates the activation of cell division and embryogenic cell formation in leaf protoplast in the presence of auxin (Otvos et al., 2005). Copper-induced morphological responses are also mediated by auxin and NO in *Arabidopsis* seedlings (Peto et al., 2011). All these previous evidences led to investigate the possible interplay between these two signal molecules. Briefly, in the case of auxin, its perception is mediated by the F-box protein TIR1 (transport inhibitor response1) and the related proteins, AUXIN SIGNALING F-BOX proteins (AFBs; Dharmasiri et al., 2005; Kepinski and Leyser, 2005). Auxin binding stabilizes the interaction between TIR1/AFBs and the transcriptional repressor proteins, auxin/indole-3-acetic acid (Aux/IAA) causing a rapid proteasomal degradation of them (Gray et al., 2001). Then, Aux/IAA degradation results in the activation of transcriptional responses with the concomitant impact in plant growth and development (Tan et al., 2007). In an attempt to study the possible mechanism by which NO might regulate auxin signaling, *S*-nitrosylation of auxin receptor was analyzed. *S*-nitrosylation of TIR1 was demonstrated by Terrile et al. (2012). This redox-based modification enhances the efficiency by which TIR1 interacts with Aux/IAAs facilitating their degradation and modulating auxin signaling during root growth in *Arabidopsis* seedlings (**Figure 2**). Particularly, Cys-140 is a critical residue for TIR1–Aux/IAA interaction and TIR1 function. *S*-nitrosylation of TIR1 represents an efficient mechanism by which NO might enhance sensitivity and/or ligand selectivity. Furthermore, NO modulation of auxin signaling is more complex since a combinatorial TIR1/AFB–Aux/IAA co-receptor system could be assembled, contributing to the versatility of auxin response (Calderon Villalobos et al., 2012). However, cellular effectors of denitrosylation remain to be explored. Recently, Correa-Aragunde et al. (2013) described a new convergence where auxins are thought to influence *S*-nitrosylation/denitrosylation balance in *Arabidopsis* roots. The antioxidant enzyme, APX1 is an *S*-nitrosylation target and auxin induces denitrosylation and partial inhibition of its activity (Correa-Aragunde et al., 2013). These authors postulated that an auxin-regulated balance of APX1 *S*-nitrosylation/denitrosylation state contributes to a fine-tuned control of reactive oxygen species (ROS) that finally impacts on root architecture and development. Recent studies have pointed out the correlation between ROS and auxin homeostasis in signal transduction during plant development and stress response (Tognetti et al., 2012). In this direction, Bashandy et al. (2010) also highlighted the intercellular redox status as a critical parameter determining plant development through modulation of auxin signaling, transport, and homeostasis. Although our knowledge about auxin and NO is currently being born, most probably *S*-nitrosylation/denitrosylation is of great impact throughout to interlink these two molecules along plant lifecycle.

Plant hormones CKs are well known for their ability to promote cell division and they are associated with growth and development, including lateral root formation and nodulation in legumes (Gonzalez-Rizzo et al., 2006; Murray et al., 2007; Tirichine et al., 2007), circadian rhythms (Salome et al., 2006), and shoot and root development (Werner and Schmulling, 2009). Recently, NO-mediated CK functions have been associated to cell proliferation and meristem maintenance in *Arabidopsis* (Shen et al., 2013). CKs are perceived and mediated by a multi-step two-component circuit through a histidine and aspartate phosphorelay (Muller and Sheen, 2007). CKs regulate their signals through a variety of mechanisms, such as modulating transcription, controlling phosphorelay and regulating protein localization and stability (To and Kieber, 2008). In a recent report, Feng et al. (2013) demonstrated that NO represses CK signaling by inhibiting the phosphorelay activity through *S*-nitrosylation. Interestingly, the authors showed that NO-overproducing mutants, *nox-1* (*NO overproducer1*) and *gsnor1–3* do not respond to CK-induced shoot regeneration in *Arabidopsis* explants. Moreover, *gsnor1–3* has a substantial reduction on the expression of the primary response regulator genes (ARRs) for CK signaling. Centrally, by the use of an *in vivo* biotin-switch assay, it was demonstrated that the histidine phosphotransfer protein AHP1 is *in planta* *S*-nitrosylated under normal growth conditions. Cys-115 was proposed as an *S*-nitrosylated residue. Comprehensively, AHP1 *S*-nitrosylation compromises CK action revealing again, a mechanism through which CK signaling components perceive and integrate a redox signal in the regulation of plant growth and development (**Figure 2**). Although several lines of evidence support the involvement of NO in CK signaling (Carimi et al., 2005; Tun et al., 2008), other works claim an opposite effect of NO in CK action (Werner et al., 2003; Riefler et al., 2006; Xiao-Ping and Xi-Gui, 2006). Much more recently, a direct interaction between NO and CK has been also described (Liu et al., 2013). In summary, NO roles could be of the most varied because in addition to its own action it meets specific cellular functions according to the target molecules amending within the routes of hormonal regulation in plant cells.

## CONCLUDING REMARKS AND PERSPECTIVES

NO is a fascinating molecule with remarkable feats and properties to modulate signaling pathways in biological systems. The bioactivity of NO is high enough for it to occur in a wide variety of biochemical circumstances. *S*-nitrosylation/denitrosylation is currently accepted as critical redox-mediated regulation processes in plant cells. Certainly, *S*-nitrosylation could be a possible mechanism by which NO impacts on plant hormonal regulation by modulating hormone biosynthesis, perception, transport, and/or degradation. Clearly, multiple layers of interactions may be involved in the plant hormones and NO cross-talks, depending on complex biological and biochemical scenarios in cells. However,nowadays fragmented studies on its *in vivo* function hamper our thorough understanding on hormone–NO cross-talking. Probably, high-throughput genetic and protein-based approaches in combination with a deeper understanding on the basic structure/function relationships of NO generating systems will shed light on this scientific riddle.

## Conflict of Interest Statement

The authors declare that the research was conducted in the absence of any commercial or financial relationships that could be construed as a potential conflict of interest.
